# Supplementary material for: A clinical audit on longer-term stroke management as a specific service in a primary care setting: Assessing adherence of service and clinical parameters
Source: PLOS Glob Public Health. 2024 Dec 4;4(12):e0002759. doi: 10.1371/journal.pgph.0002759 (PMC11616876; doi:10.1371/journal.pgph.0002759)
Supplement: S1 Appendix — (DOCX) [file pgph.0002759.s001.docx]

# APPENDICE 1

**Check-list form used in the audit**

| **Anonymize ID number** |  | | | | | | |
| --- | --- | --- | --- | --- | --- | --- | --- |
| **RN** |  | | | | | | |
| **Item** |  | | | | | Remarks | |
| - - 1. **demographically details** | | | | | | | |
| Age (years) |  | | | | |  | |
| Gender | Male | | | | |  | |
|  | Female | | | | |  |  |
| Ethnicity | Malay | | | | |  | |
|  | Chinese | | | | |  |  |
|  | Indian | | | | |  |  |
|  | Others | | | | |  |  |
| Education level | No | | | | |  | |
|  | Primary school | | | | |  |  |
|  | Secondary school | | | | |  |  |
|  | College/university | | | | |  |  |
|  | NA | | | | |  |  |
| Occupational status  Unemployed/retiree | Fixed salaried employee | | | | |  | |
|  | Self-employed | | | | |  |  |
|  | Unemployed/retiree | | | | |  |  |
| Living arrangements | Alone | | | | |  | |
|  | Family | | | | |  |  |
|  | Friends | | | | |  |  |
|  | Nursing home | | | | |  |  |
| - - 1. **Clinical profiles** | | | | | | | |
| Age of stroke (years) |  | | | | |  | |
| Smoking status | Yes | | | | |  | |
|  | No | | | | |  |  |
| Stroke episode | First | | | | |  | |
|  | Subsequent | | | | |  |  |
|  | Unsure | | | | |  |  |
| Stroke subtype | Hemorrhagic | | | | |  | |
|  | Ischemic | | | | |  |  |
|  | Mixed | | | | |  |  |
|  | Unspecified | | | | |  |  |
| Diabetes mellitus | Yes | | | | |  | |
|  | No | | | | |  | |
| Hypertension | Yes | | | | |  | |
|  | No | | | | |  |  |
| dyslipidaemia | Yes | | | | |  | |
|  | No | | | | |  |  |
| Neurorehabilitation program (after acute stroke period) | Yes | | | | |  | |
|  | No | | | | |  |  |
| Physiotherapy (after acute stroke period) | Yes | | | | |  | |
|  | No | | | | |  |  |
| Occupational therapy (after acute stroke period) | Yes | | | | |  | |
|  | No | | | | |  |  |
| Speech & language therapy (after acute stroke period) | Yes | | | | |  | |
|  | No | | | | |  |  |
| - - 1. **LTSC Post-stroke Checklist** | | | | | | | |
|  | Assessed/Mentioned by the healthcare provider | | | | | | |
|  | Yes | | | No | | | |
| 1. Secondary prevention – medical advice/medications |  | | |  | | | |
| 1. Physical    1. ADL – mRS, Modified Barthel Index, IADL    2. Mobility    3. Spasticity/stiffness/contracture    4. Pain    5. Incontinence – bowel/bladder |  | | |  | | | |
| 1. Speech    1. Communication |  | | |  | | | |
| 1. Cognition assessment    1. Memory – ECAQ/MMSE    2. Emotional functioning – TQWHQ/PHQ |  | | |  | | | |
| 1. Life after stroke    1. Leisure activities    2. Driving    3. Back to work |  | | |  | | | |
| 1. Relationship - Personal/Family |  | | |  | | | |
| 1. Fatigue |  | | |  | | | |
| 1. Other challenges |  | | |  | | | |
| 1. Nutritional |  | | |  | | | |
| 1. Financial – self, social welfare |  | | |  | | | |
| 1. Community resources |  | | |  | | | |
| - - 1. **Clinical outcome parameters** | | | | | | | |
|  | **Initial visit/baseline** | | **Latest visit** | | | | **Remarks** |
| Blood pressure (mm Hg) |  | |  | | | |  |
|  |  | | Achieved < 140/90 mm Hg? | | | |  |
|  |  |  | Yes | | No | |  |
| Systolic blood pressure (mm Hg) |  | |  | | | |  |
| Diastolic blood pressure (mm Hg) |  | |  | | | |  |
| LDL Cholesterol (mmol/L) |  | |  | | | |  |
|  |  | | If non-diabetic, achieved < 1.8 mmol/L? | | | |  |
|  |  |  | Yes | | No | |  |
|  |  |  | If diabetic, achieved < 1.4 mmol/L? | | | |  |
|  |  |  | Yes | | no | |  |
| HbA1c (%) |  | |  | | | |  |
|  |  | | Achieved <7%? | | | |  |
|  |  |  | Yes | | No | |  |
| Weight (kg) |  | |  | | | |  |
| Smoking | Yes | No | Yes | | No | |  |
