# Supplementary material for: A clinical audit on longer-term stroke management as a specific service in a primary care setting: Assessing adherence of service and clinical parameters
Source: PLOS Glob Public Health. 2024 Dec 4;4(12):e0002759. doi: 10.1371/journal.pgph.0002759 (PMC11616876; doi:10.1371/journal.pgph.0002759)
Supplement: Attachment — Submitted filename: PLOS review.docx [file pgph.0002759.s002.docx]

In the article, Mostapha et al. performed retrospective audit and published dataset to investigate the services for longer-term stroke care compared to the established standardized criteria. This article is not suitable for publication in its current form for the following reasons:

1. The objectives need more precise definition, and the paper's logical flow requires improvement. In the introduction, authors should clarify the existing gaps in the field, explain how their research addresses these deficiencies, and potentially fills the gap of unmet needs. Subsequently, each subsection within the results section should commence with a clear statement of purpose, ensuring a seamless transition from the preceding section. Consistent data interpretations and conclusions for each segment are essential within the results section.
2. The article contains a few grammatical errors and it would benefit from language editing.
3. The methods lack detailed descriptions. Figure legends are missing crucial information, such as the type of samples and experiments conducted.
4. The study involves the utilization of various databases and algorithms. It is essential for the authors to provide concise descriptions of each resource, outline the objectives of the analysis, and justify the reasons behind choosing these particular resources and methodologies.

Additional points:

1. The introduction should be served as a review of what is known prior to this current work. I suggest reviewing on current standardized tools, unmet needs of long-term stroke patient management and identifying risk factors of post-stroke complications. What are the current diagnosis limitations and challenges and how your work can help close the knowledge gap? I suggest listing some specific examples. Highlighting these topics will show the importance of your work.
2. The authors should seek for better representations for some of the figures, since some are not informative (see more comments below). The font sizes are too small in some figures.
3. For results, table 4 and 5, should be presented in better ways. For example, you can show adherence of services parameters on Post-stroke Checklist using bar plots and arrange by descending order.
1. For table 6, I suggest Please highlight the items that are significantly different when comparing initial and latest clinical parameter. Is P<0.05 the cut off for significance? I noticed weight is also one parameter, does weight loss indicate better post-stroke care or weight gain? Please specify this in the result sections.
5. I suggest include a flow chart for data selection.
6. In writing the result section, the authors should always provide some transitions from the previous section, and state their objectives first. For example, “having seen X, we next set out to understand the difference between initial and latest clinical parameters of patients attending LTSC. We perform Y, which showed Z.”
7. It would be helpful to include some future directions based on current study. For example, how would you expect to improve adherence to long-term stroke care. What are the current limitations for not adopting this care service vs. standardized?
