# Supplementary material for: A clinical audit on longer-term stroke management as a specific service in a primary care setting: Assessing adherence of service and clinical parameters
Source: PLOS Glob Public Health. 2024 Dec 4;4(12):e0002759. doi: 10.1371/journal.pgph.0002759 (PMC11616876; doi:10.1371/journal.pgph.0002759)
Supplement: Attachment — Submitted filename: Response and corrections - Global Plos One NAA (1).docx [file pgph.0002759.s003.docx]

**Dear Editor	Date: 17th March 2024**

Herewith are the changes and corrections of the manuscript ‘**A Clinical Audit on Longer-Term Stroke Management as A Specific Service in A Primary Care Setting’** based on reviewers’ comments.

The texts in brown are the addition to the manuscript whereas the underlined texts are those removed from the manuscript

Thank you for your kind consideration

| **Items** | **Comments/suggestions** | **Response/Rebuttal** |
| --- | --- | --- |
| **FIRST REVIEWER** | | |
| 1. | In the background in the 3rd line: what part of the population the prevalence reported applies to? | Statement has been clarified to “The National Health Mobility Survey (NHMS) has reported increased stroke prevalence in Malaysia from....”   1. ^11.^ |
| 2. | Methods - Statistical Section: please specify what is being compared with the t-test and McNemar | Statement has been clarified to “The Student's Paired t-test and McNemar tests were used for the inferential analysis of the outcome of the clinical parameters. |
| 3. | 1.Results 3rd: We discovered that 18% (29/161) of patients were removed due to missing data. This must be described and explained in the methods. Moreover, you remained with 113 patients, so 161 - (113 + 29) = 19. What happened to these 19?  2.What kind of missing affects the 29? I am very concerned that the remaining data has no missing data (except for education), making the whole situation really good. Missing/completeness of data should be a component of auditing as well therefore, unless clearly explained, those 29 should be included in the analysis. | It has been clarified with an additional paragraph on the process of data collection and statistical analysis.  1. “**Process of data collection & Statistical analysis:** All data were obtained from the patients’ medical records that were kept in the clinic. All the records were kept in a manual filing system in the record area. During the audit period, patients’ lists were extracted from the LTSC registry, whereby the researcher then manually searched the records in the filing area. Files were reviewed, and data were extracted and entered into the prepared proforma. Data were entered anonymously to maintain patients’ confidentiality. Patient’s data were classified as incomplete or not-fit for analysis should there be no entry during the period of audit or missing more than 30% of the required data in the proforma”.  2.Missing data in the results section has been clarified, in tally with the explanation in the methods section: “A total of 161 patients registered in the LTSC census, with 19 patients excluded at initial screening as they did not attend any session in 2022(10 patients lost to follow-up and 9 died in the year of audit). A total of 142 patients were included for analysis after fulfilling the inclusion criteria. After reviewing the exclusion criteria, 29 were excluded due to irretrievable medical records or missing data, thus leaving a total of 113 patients for audit”. |
| 4. | Table 2:  - for age, indicate the unit of measurement, and report also the median and the IQR)  - indicate that these characteristics were collected at entry, correct?  Table 3:  - for the age of stroke, indicate the unit (years?), and report also the median and the IQR  - Merge table 2 and table 3. There are so many tables here. I think PGPH allows a maximum of 5 tables | -median and IQR  -Table 2 and Table 3 have been merged as now Table 2 (Table 2: Biodata and Clinical Profiles of Patients (N: 113) |
| 5. | Table 5:  - For blood pressure (BP < 140/90 mmHg), there is a 64.6% below the standard 70%. Why is this considered achieved? I am not disagreeing/agreeing. I suggest clarifying the criteria for such a decision in the methods. | Table 5 now table 4  Has been clarified in the methodology section  “The individualized standard performance level was based on the Quality Assurance (QA) indicator within the Malaysian National Diabetes Registry Report (10) and Al-Salti et al. (12) and as follows: 70% for BP, 30% for HbA1c, and 45% for LDL-C. The standard performance levels for each clinical outcome parameter can be found in Table 5”. |
| 6. | Table 6:  - this analysis requires a cohort followed. A hallmark of a cohort is follow-up time. The reported mean changes here suggest that the amount of follow-up time for every patient is similar. Is this a valid assumption? For example, one patient may take 6 months to reach the same change as another would require just 3. So, a change per month analysis would be better. | Table 6 now Table 5  Has been clarified in the text  “Table 5 illustrates the changes of clinical parameters over the 6 months period (of two regular LTSC appointments)”. |
| 7. | Table 7:  - Good that authors conducted a McNemmar test (the issue of follow-up time is OK)  - You could report an odds ratio. Add 0.5 in all cells to deal with the zero. | After reviewing the necessity and relevance of this segment, Table 7 was dropped from the manuscript as it did not carry much value to the manuscript. We added the change in smoking status as text in the results segment  “Regarding the comparison of smoking status, there was a reduction of post-stroke patients who smoke, from 15 patients (13.3%) to only eight patients (7.1%) who smoked during the most recent follow-up, which is approximately 50% from the initial consultation.” |
| 8. | In the discussion:  - remove the p-values. If you want to keep them, please report accurate p-values, not just p<0.05. | The p values had been removed as suggested |
| 9. | The first and second paragraphs disregard an important bias when comparing cross-sectional. For your sample, you included only people who survived to be included in your sample. If those who have a stroke and die tend to be older than the ones in your sample, then you may have a biased mean age at stroke. Please reformulate your statement. | The confusion has been clarified  "The clinical profiles of patients who received care at the LTSC facility in the year of audit revealed that the average age of first stroke was 57.68 years ± 12.04. We acknowledged that the mean age could be higher as we excluded those who missed appointments and those who died. Nonetheless it portrayed the recent trend of stroke in SouthEast Asia that affects the younger population compared to the global average for stroke, which was reported as 64.4 years (18). This finding mirrored the current scenario in Malaysia, where the recent mean age for stroke prevalence in Malaysia is 54.5 - 62.6 years old (2).” |
| 10. | The objectives need more precise definition, and the paper's logical flow requires improvement. In the introduction, authors should clarify the existing gaps in the field, explain how their research addresses these deficiencies, and potentially fills the gap of unmet needs. | Points addressing the objectives of the audit and existing gaps in current literature have been added to the manuscript  “The last two decades have seen advancement in research with emerging evidence on acute stroke and rehabilitation, nonetheless, there are still gaps in finding the ideal management for stroke patients after hospital discharge. Thus, as stroke management is complex and involves a long period of time, it is suggested that stroke management should go beyond hospitals, reaching towards comprehensive multidisciplinary management namely, further rehabilitation, primary care, nursing and community stroke care services (4)  The concept of comprehensive stroke services had come into attention in the late 1990s after the realization that stroke patients that were discharged from acute care were in need of follow-up management for them to have better quality of life and reduce the risk of recurrences (5). These services varied from region to region, adapting to factors namely; stroke services accessibility, patients’ needs, existing resources, stroke team members’ expertises and national policies and financial support on stroke care. A systematic review on unmet needs on stroke survivors found that the unmet needs ranged from health-related needs to re-integration into the community. Stroke survivors reported unmet needs in physical, emotional and cognitive functions, and resources in looking for support and social participation (6). The authors suggested that stroke patients in the community would benefit from targeted stroke care services that addressed the various unmet needs after stroke using standardized assessment tools, thus providing an objective and comprehensive care to the patients and families. They also emphasized on the needs for research that focus on the requirement in developing primary care-based stroke with a targeted approach to long-term support”  References added to this segment  4.Noor Azah Abd Aziz, Nor Shahrina Mohd Zawawi, Wan Nur Sabrina Wan Manshol, Saw Mei Mei, Mohd Fairuz Ali. (2022). Communication: The Need for Inclusive Algorithm and Multi-Disciplinary Approach in Managing Long-Term Stroke Care Patients the Community, Focusing on Primary Care. Journal of Family Medicine and Health Care, 8(3), 56-61. https://doi.org/10.11648/j.jfmhc.20220803.12  5. Langhorne P. Developing comprehensive stroke services: an evidence-based approach. Postgrad Med J. 1995 Dec;71(842):733-7. doi: 10.1136/pgmj.71.842.733. PMID: 8552537; PMCID: PMC2398274.  6. N.S.M. Zawawi, N.A. Aziz, R. Fisher, K. Ahmad, M.F. Walker, The Unmet Needs of Stroke Survivors and Stroke Caregivers: A Systematic Narrative Review, Journal of Stroke and Cerebrovascular Diseases, Volume 29, Issue 8, 2020, 104875, ISSN 1052-3057, https://doi.org/10.1016/j.jstrokecerebrovasdis.2020.104875) |
| 11. | The methods lack detailed descriptions. Figure legends are missing crucial information, such as the type of samples and experiments conducted. | Amended and added into the manuscript  This study |
| 12. | The study involves the utilization of various databases and algorithms. It is essential for the authors to provide concise descriptions of each resource, outline the objectives of the analysis, and justify the reasons behind choosing these particular resources and methodologies | Amended and added into the methodology section  The individualized standard performances level was based on the Quality Assurance (QA) indicator within the Malaysian National Diabetes Registry Report (10) and Al-Salti et al. (12) as follow: 70% for BP, 30% for HbA1c, and 45% for LDL-C. The standard performance levels for each clinical outcome parameter can be found in Table 4. |
| **ADDITIONAL POINTS** | | |
| 1. | .The introduction should be served as a review of what is known prior to this current work. I suggest reviewing on current standardized tools, unmet needs of long-term stroke patient management and identifying risk factors of post-stroke complications. What are the current diagnosis limitations and challenges and how your work can help close the knowledge gap? I suggest listing some specific examples. Highlighting these topics will show the importance of your work | Has been amended with additional input in Paragraph 2 in Background section  “Living with stroke………..primary care-based stroke with a targeted approach to long-term support” |
| 2. | For results, table 4 and 5, should be presented in better ways. For example, you can show adherence of services parameters on Post-stroke Checklist using bar plots and arrange by descending order. | Table now (3 and 4) are maintained in the current form with corrections, as we believe it is more informative to the readers |
| 3. | For table 6, I suggest Please highlight the items that are significantly different when comparing initial and latest clinical parameter. Is P<0.05 the cut off for significance? I noticed weight is also one parameter, does weight loss indicate better post-stroke care or weight gain? Please specify this in the result sections. | Thank you for the comments   1. Table (now) 5 has been amended, with significant p value has been highlighted 2. The weight parameter has been deleted as it did not bring any additional point to the manuscript. |
| 4. | I suggest include a flow chart for data selection | Flow chart is not added as it exceeded the word counts for the manuscript |
| 5. | In writing the result section, the authors should always provide some transitions from the previous section, and state their objectives first. For example, “having seen X, we next set out to understand the difference between initial and latest clinical parameters of patients attending LTSC. We perform Y, which showed Z | The results section has been amended to highlight the findings and make better of the results’ sequences. |
| 6. | It would be helpful to include some future directions based on current study. For example, how would you expect to improve adherence to long-term stroke care. What are the current limitations for not adopting this care service vs. standardized? | Amended in text  Overall, the results of this audit highlighted the possibility of providing structured stroke care services that can be embedded within the existing primary care services. The use of the standardised Post-stroke checklist and clinical parameters are shown to be useful and practical in managing stroke patients in the community, majority with various complex issues and cardiovascular risk factors which in need of comprehensive care such as this. As the service is within the primary care jurisdiction, it could be added into the clinic’s services effortlessly. In conclusion, these findings support the value of the long-term stroke care services provided in the primary care setting and provide a foundation for further enhancements and future evaluations. |

Corresponding author (on behalf of all authors)

**Noor Azah Abd Aziz**
